# A rare variant of the mtDNA HVS1 sequence in the hairs of Napoléon's family

**DOI:** 10.1186/2041-2223-1-7

**Published:** 2010-10-04

**Authors:** Gérard Lucotte

**Affiliations:** 1 Institute of Molecular Anthropology, Paris, France

## Abstract

This paper describes the finding of a rare variant in the sequence of the hypervariable segment (HVS1) of mitochondrial (mtDNA) extracted from two preserved hairs, authenticated as belonging to the French Emperor Napoléon I (Napoléon Bonaparte). This rare variant is a mutation that changes the base C to T at position 16,184 (16184C→T), and it constitutes the only mutation found in this HVS1 sequence. This mutation is rare, because it was not found in a reference database (*P *< 0.05). In a personal database (M. Pala) comprising 37,000 different sequences, the 16184C→T mutation was found in only three samples, thus in this database the mutation frequency was 0.00008%. This mutation 16184C→T was also the only variant found subsequently in the HVS1 sequences of mtDNAs extracted from Napoléon's mother (Letizia) and from his youngest sister (Caroline), confirming that this mutation is maternally inherited. This 16184C→T variant could be used for genetic verification to authenticate any doubtful material and determine whether it should indeed be attributed to Napoléon.

## Introduction

Genetic identification of old biological specimens is often limited to the analysis of short, degraded DNA fragments, but the development and application of comprehensive DNA testing for identification of old forensic or historical samples is of considerable interest. In the early 1990 s, forensic investigation of a grave found in Russia [1] suggested that the human remains it contained were those of members of the imperial Romanov family, specifically Emperor Nicholas II, his wife Empress Alexandra and their children, together with three servants and a physician, who were all killed during the Russian Civil War in 1918.

Owing to its high copy number, its rapid rate of evolution, and its haploid and maternal mode of inheritance, mitochondrial (mtDNA) has a number of advantages over autosomal DNA markers for the identification of human remains [2]. The high copy number, with several hundred mtDNA molecules per cell, means that with older remains there is a greater likelihood of success in analyzing mtDNA as opposed to autosomal DNA, simply because mtDNA is more abundant. The mtDNA control region, which includes the origin of H strand replication, the displacement (D) loop and both origins of transcription, is the most variable region of the human mitochondrial genome [3]. Most polymorphisms are concentrated in two hypervariable segments (HVS1 and HVS2), one of which encompasses the origin of replication, while the other lies within the D loop itself.

The aim of the present study was to investigate if there are variants in the HSV1 mtDNA sequence of the French Emperor Napoléon I (Napoléon Bonaparte; 1769-1821) compared with the Anderson human genome sequence [3].

A rare variant was found in the HVS1 sequence of the mtDNA extracted from preserved hairs, authenticated as belonging to Napoléon. Subsequently, the same rare variant was found in the HSV1 sequences of mtDNAs extracted from hair samples authenticated as belonging to Napoléon's mother (Letizia) and youngest sister (Caroline).

## Materials and methods

### Preserved hairs

**Figure 1:**
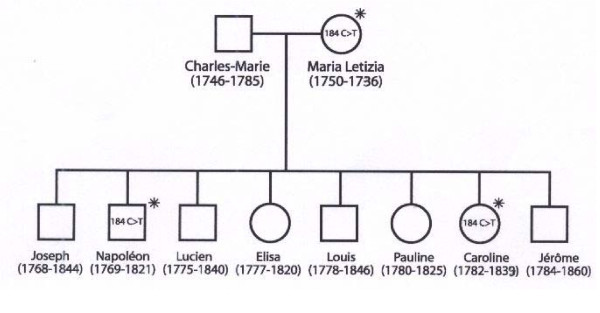
**Pedigree of the close family of Napoléon I**. The 16184C→T variant (abbreviated to 184C→T) in the HVS1 sequences of Letizia, Napoléon and Caroline is indicated.

**Figure 2:**
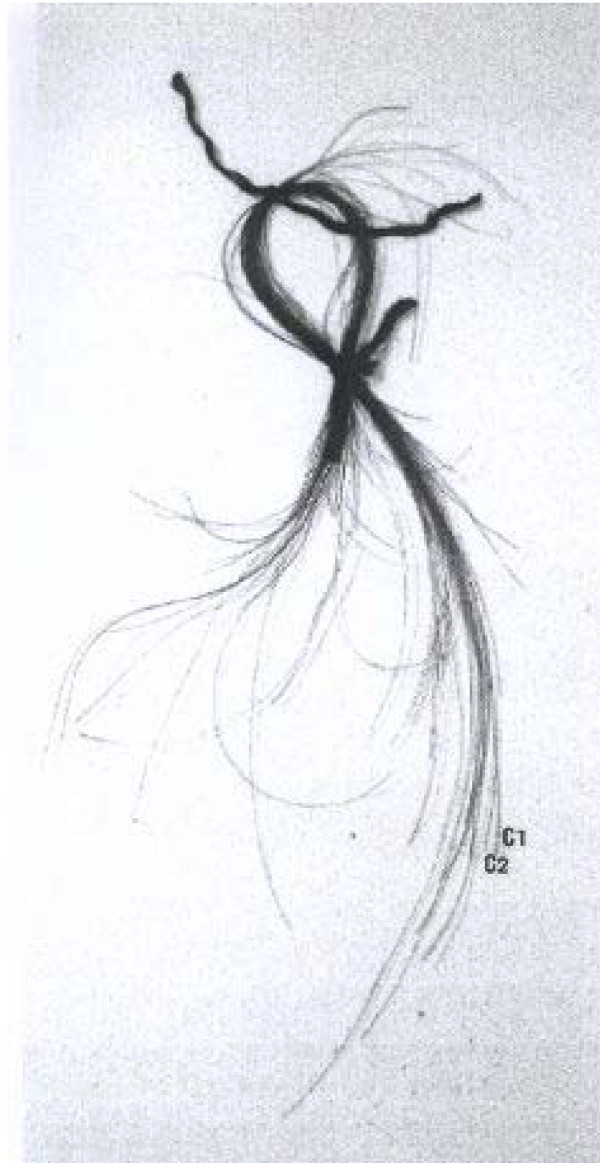
**The hairs of Napoléon's I, contained in the reliquary Vivant-Denon**. The samples C1 and C2 were removed for analysis.

Three samples of preserved hairs were analyzed in this study, originating from three members of the Bonaparte family (Figure 1). The first was a lock of Napoléon's own hair (Figure 2), kept in the reliquary Vivant-Denon (deposited in the Bertrand Museum of Châteauroux), which is authenticated by Napoléon's signature on a letter also kept in the reliquary, addressed to Vivant-Denon. Two hair specimens (C1 and C2) were taken for analysis. The second hair sample was from Letizia, preserved in a silver box deposited in the reserve of the Bertrand Museum and authenticated by a certificate written by the two physicians who had taken part in the mummification of Letizia's body; several hairs were taken from a lock of hair contained in the silver box, and one of these hairs was used for the present analysis. The third sample, Caroline's hair, came from a private collection, and was authenticated by the current proprietor.

### Working conditions

The pre- and post-PCR steps were carried out in physically separated laboratories to avoid crosscontamination. All surfaces and instruments were treated with chloride bleach, and all plasticware and solutions underwent UV irradiation. All workers wore gloves and facemasks throughout the entire procedure. Disposable gamma-irradiated pipette tips (Diamond; Gilson Inc., Middleton, MA, USA), sterile microcentrifuge tubes (Biopur; Eppendorf, Hamburg, Germany) and gamma-irradiated PCR tubes (Molecular BioProducts Inc., San Diego, CA, USA) were used.

To control for any potential DNA contamination, each PCR included two negative controls: a PCR control and an extraction control (the latter being prepared at the start of the extraction to check for contamination during the extraction procedure).

### DNA extraction

For Napoléon's samples, the initial DNA extraction and HVS1 sequencing were performed on hair sample C1, and the results later confirmed with the second hair sample, C2. For both samples, three independent PCR amplifications were performed and two replication runs then performed to verify the sequence. Similarly, DNA extraction and HVS1 sequencing were performed on one hair each from Letizia and Caroline.

In each case, DNA was extracted from the hair fragment specimen using a standard method (0.5 M EDTA, sarcosyl 20% and proteinase K 10 mg/ml), and purified using a commercial kit (NucleoSpin^® ^Kit; Macherey-Nagel, Duren, Germany), in accordance with the manufacturer's instructions with some modifications.

Each DNA extraction was performed independently in an isolated laboratory (previously used mainly for work with human DNA), dedicated to working with ancient DNA.

### HVS1 PCR

PCR procedures were performed in sterile PCR hoods in accordance with standards for ancient DNA work, with regular decontamination measures and all precautions taken to avoid any risk of contamination by modern DNA molecules. The mtDNA genomic sequence interval for HVS1 from positions 15,991 to 16,390 was amplified by PCR with primers F15971 (5'-TTAACTCCACCATTAGCACC-3') and R16410 (5'-GTCCCTTGACCACCATCCTC-3').

For each PCR, the DNA extract from hair specimen was amplified in a 12.5 μl reaction mixture (2 mM MgCl2, 50 mM KCl, 10 mM Tris/HCl pH 9, 0.1% Triton X-100, 0.2 mM each dNTP, 0.1 μM each primer and 2.5 U of DNA polymerase (Ampli Taq Gold; Applied Biosystems, Foster City, CA, USA)). The amplification was carried out with an initial denaturation step at 95°C for 6 min, followed by 35 cycles 95°C for 1 min, 55°C for 1 min, and 72°C for 1 min.

### DNA sequencing and alignment

PCR products were purified from agarose gel (QIAQuick PCR Purification Kit; Qiagen, Valencia, CA, USA). Both strands of all the amplified mtDNA fragments eluted from the agarose gel slices were directly sequenced (Big Dye Terminator Cycle Sequencing Kit; Applied Biosystems) and separated (ABI PRISM 3130 × l Genetic Analyzer; Applied Biosystems). The sequences obtained were aligned against the Revised Cambridge Reference Sequence [4] to identify the presence of polymorphic sites. SeqScape software (Applied Biosystems) and Clustal analysis http://www.clustal.org were used for pairwise alignment.

## Results

**Figure 3:**
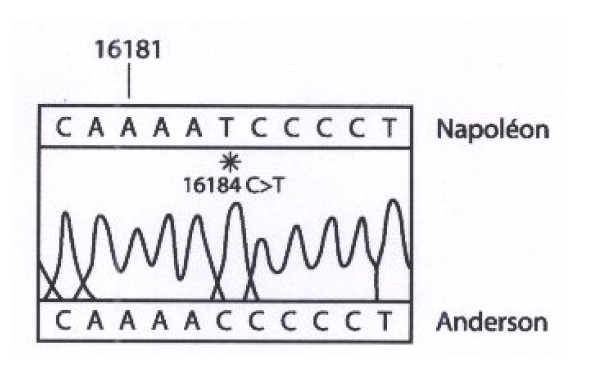
**The 16184C→T variant**. At position 16,184 of the Anderson sequence, the base C is replaced by T.

The sequence obtained from Napoléon's hair sample matched the Anderson sequence, except for the position 16,184 where the C was replaced by a T (Figure 3), named as variant site C16184T (or mutation 16184C→T). This variant was also the only mutation of the HVS1 sequence found in the mtDNA extracted from the C2 sample.

To investigate the frequency of the 16184C→T mutation in the general population, we performed a search of the US Federal Bureau of Investigation site 'mtDNA Search', which contains information on 4,839 mtDNA sequences (1,674 of them of White ethnic origin). The HVS1 sequence with the 16184C→T variant was not represented in this database, thus giving a frequency of 16184C→T in this database of < 0.05%. In a subsequent search of a public database, EMPOP http://www.empop.org, containing data on 4,475 individuals, the 16184C→T mutation was found to be present in three individuals of West Eurasian origin, giving a frequency of 0.0007%. In a personal database comprising about 37,000 control region records (Dr M. Pala, personal communication), the HSV1 sequence with the single mutation 16184C→T was also present in three samples (one from Crete and two from Italy), giving a mutation frequency for 16184C→T of 0.00008%. All three samples belonged to the mtDNA haplogroup H. The mutation at position 16184 was very recently found [5] in one Italian HVS1 sequence (but this sequenced sample harbors several additional mutations, indicating that it belongs to haplogroup U5b3). Napoléon's HVS1 mtDNA does not harbor these additional mutations, and there is evidence that this DNA belongs to haplogroup H (Pr A. Torroni, personal communication).

In the subsequent analyses, the 16184C→T mutation was also found to be the only variant in the HVS1 sequences of the mtDNAs extracted from the hair samples of Letizia and Caroline.

The fact that these three independent hair samples all had the same sequence is a valuable check on the authenticity of the results. We also know that the main technician (N.C.) involved in this research has the mtDNA haplogroup H, but does not have the mutation in her HVS1 sequence (excluding the possibility of crosscontamination).

## Discussion

In this study, a rare variant (16184C→T) of the HVS1 sequence was found in mtDNA extracted from preserved hair samples taken from the Emperor Napoléon I. As the DNA was mitochondrial, the mutation was passed down from the mother (Letizia) to all of her descendants (only two, Napoléon and his sister Caroline, having been tested in the present study). There is therefore a high probability that this mutation is present in all living women descendant from Napoléon's mother by maternal lineage.

We found that the variant 16184C→T is extremely rare, having a frequency of << 0.01% in three databases tested. So, because of its rarity, the detection of this mutation constitutes an ideal tool for authentication of other body samples (e.g. hair, bone, skin...) purported to be those of Napoléon I, which are present in various museums and in some private collections around the world. A huge number of locks of hair (too many to be possible) purporting to those of Napoléon, especially those supposedly collected during his exile in St Helena (Sainte-Hélène) and after his death, are known to be among these relics. A simplified DNA method (protocol available on demand) based on the present results could be used as a preliminary genetic test (requiring only one portion of hair for each lock) to establish if these locks really are those of Napoléon.

In addition, there is currently widespread controversy about the remains present in the crypt of Les Invalides in Paris [6]. Is the body contained in the porphyr sarcophagus in this museum that of the Emperor, or is it possibly that of another man? If the latter, might it be the body of one of the Emperor's servants? Exhumation of the corpse contained in the sarcophagus (or, more simply, examination of a portion of frontal skin of the Emperor contained in a medallion) would permit extraction of genetic material and genetic analysis of the HVS1 sequence of the mtDNA, and should thus provide answers to these questions.

## Competing interests

The author declares that they have no competing interests.
